# Topology and Organization of the *Salmonella typhimurium* Type III Secretion Needle Complex Components

**DOI:** 10.1371/journal.ppat.1000824

**Published:** 2010-04-01

**Authors:** Oliver Schraidt, Matthew D. Lefebre, Matthias J. Brunner, Wolfgang H. Schmied, Andreas Schmidt, Julia Radics, Karl Mechtler, Jorge E. Galán, Thomas C. Marlovits

**Affiliations:** 1 Research Institute of Molecular Pathology, Vienna, Austria; 2 Institute of Molecular Biotechnology GmbH, Austrian Academy of Sciences, Vienna, Austria; 3 Yale University School of Medicine, Section of Microbial Pathogenesis, Boyer Center for Molecular Medicine, New Haven, Connecticut, United States of America; 4 CD-Laboratory for Proteome Analysis, Vienna, Austria; The Rockefeller University, United States of America

## Abstract

The correct organization of single subunits of multi-protein machines in a three dimensional context is critical for their functionality. Type III secretion systems (T3SS) are molecular machines with the capacity to deliver bacterial effector proteins into host cells and are fundamental for the biology of many pathogenic or symbiotic bacteria. A central component of T3SSs is the needle complex, a multiprotein structure that mediates the passage of effector proteins through the bacterial envelope. We have used cryo electron microscopy combined with bacterial genetics, site-specific labeling, mutational analysis, chemical derivatization and high-resolution mass spectrometry to generate an experimentally validated topographic map of a *Salmonella typhimurium* T3SS needle complex. This study provides insights into the organization of this evolutionary highly conserved nanomachinery and is the basis for further functional analysis.

## Author Summary

Many Gram negative pathogens such as *Salmonella*, *Yersinia*, or *Shigella* use the type III secretion system (T3SS) to initiate infection in eukaryotic cells, resulting in well known clinical symptoms ranging from mild headaches and diarrhea to life-threatening diseases such as typhoid fever or bubonic plague. The T3SS is a highly developed macromolecular system that serves as a platform to make physical contact between host cells and pathogens and mediates the translocation of bacterial toxins (effector proteins) into eukaryotic cells. Central to the T3SS is the mega-dalton sized membrane associated needle complex, which is composed of several soluble and membrane proteins; however, their organization within the needle complex critical for proper assembly and function is unclear. Here, we use an integrated experimental approach that combines cryo electron microscopy with bacterial genetics, site-specific labeling, mutational analysis, chemical derivatization and high-resolution mass spectrometry in order to determine the topographic organization of individual components of the *Salmonella typhimurium* needle complex and define sites critical for its stability. Our study provides insights into the organization of this evolutionary highly conserved system and is the basis for further functional analysis.

## Introduction

One of the most exciting recent developments in the field of bacterial pathogenesis is the discovery that many bacterial pathogens utilize supramolecular nanomachines to deliver bacterial proteins into eukaryotic cells. These proteins, which are collectively referred to as effectors, have the capacity to modulate a variety of cellular functions including cytoskeleton dynamics, vesicle traffic, cell cycle progression and transcription. At least four types of machines capable of transporting effectors have been identified. They are known as type II, type III, type IV, and type VI protein secretion systems [1]–[5]. Arguably the best understood of these machines are the type III secretion systems (T3SS), which are essential for the virulence of several important bacterial pathogens including *Salmonella enterica*, *Shigella* spp., *Yersinia* spp., enteropathogenic strains of *E. coli*, and *Vibrio cholerae*. A central component of T3SS is the needle complex, a multiprotein structure that mediates the passage of the effector proteins through the bacterial envelope. Although this structure was initially identified in *Salmonella enterica* serovar *typhimurium* (*S. typhimurium*) [6], it has been shown to be conserved in other bacteria encoding T3SSs [7],[8]. The cylindrically shaped needle complex is composed of a multi-ring base (∼25nm in width and ∼30nm in length), associated to the bacterial envelope, and a needle-like extension that protrudes several nanometers (∼20–50nm) from the bacterial surface. The needle is anchored to the base through another substructure, the inner rod, which together with the needle filament forms a ∼3nm wide channel that serves as conduit for the proteins that travel this secretion pathway [9]. Assembly of the needle complex occurs in discrete steps that first lead to the assembly of the base substructure [10]. Once assembled, the base begins to work as a secretion machine although exclusively devoted to the secretion of the proteins required for the assembly of the inner rod and the needle. Only upon complete assembly, the secretion machine changes substrate specificity and becomes competent for the secretion of effector proteins destined for delivery into eukaryotic target cells [5]. This functional reprogramming is believed to involve significant conformational changes in the needle complex itself [11].

*S. typhimurium* encodes two different T3SS within its pathogenicity island 1 (SPI-1) and 2 (SPI-2), which in a coordinated fashion mediate bacterial uptake into and replication within epithelial cells. Previous biochemical and genetic studies have established that the SPI-1-encoded *S. typhimurium* needle complex is composed of the bacterial proteins PrgH, PrgK, and InvG, which make up the base substructure, and PrgI and PrgJ, which constitute the needle and inner rod substructures, respectively [6],[12]. Cryo electron microscopy and single particle analysis have provided a ∼17Å resolution density map of the *S. typhimurium* SPI-1-encoded needle complex [9]. Recently, atomic structures of soluble domains of protein components from needle complexes from various bacterial species have become available [13]–[15]. These studies have revealed the presence of a conserved domain within the main components of the base, which in the *S. typhimurium* SPI-1 T3SS are InvG, PrgH and PrgK. Given the fact that these three proteins apparently organize themselves in a ring-like fashion, it has been proposed that this domain may mediate the formation of these rings. Attempts have been made to dock these protein domains into the needle complex structure. However, the relatively low resolution of the available electron microscopy density map does not allow the confident placement of the atomic structures of the different protein domains without additional experimental verification. Consequently, different, and in some case mutually incompatible, locations have been proposed for various protein domains [15],[16].

In this study, we have used a combination of methods including bacterial genetics, biochemistry, mass spectrometry and cryo electron microscopy/single particle analysis to experimentally assign specific protein domains to different substructures of the needle complex. In addition, we have identified specific interaction sites among components of the needle complex, which are critical for its stable assembly. Combined, this analysis provides the first experimentally validated topographic map of different components of the needle complex of the *S. typhimurium* SPI-1 TTSS.

## Results

### InvG forms the outer rings and neck region and PrgH and PrgK form the inner rings of the needle complex

**Figure 1:**
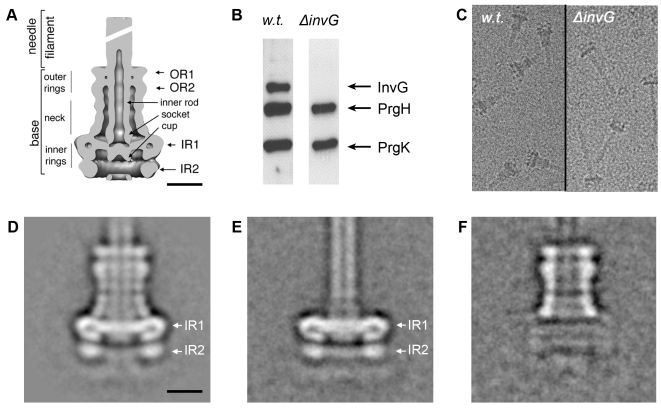
InvG forms the outer rings and neck region of the needle complex. (**A**) Cut-away view and description of individual substructures of the needle complex from *S. typhimurium*. (OR outer ring; IR inner ring) Bar = 10nm. (**B–F**) Analysis of complexes obtained from a S. *typhimuirum ΔinvG* strain: Western blot analysis (**B**), cryo electron microscopy images (**C**), and class averages of non-tilted complexes, (**D**, **E**) isolated from wild type and *ΔinvG* mutant *S. typhuimurium* strains, respectively. (**F**) Density difference between averaged images of wild type needle complexes and *ΔinvG* mutant complexes (**1F** = **1D**-**1E**), indicates the position of the outer ring substructure.

The *S. typhimurium* needle complex component InvG belongs to the secretin family, which is composed of outer membrane proteins that are associated with several secretion systems in Gram-negative bacteria. These include proteins associated with type II (e.g. *Klebsiella pneumoniae* PulD) [17] and type III (e.g. *Yersinia* spp. YscC) protein secretion systems [18], type IV pilus assembly (e.g. *Neiseria* PilQ) [19] and filamentous bacteriophage secretion (e.g. filamentous bacteriophage pIV) [20]. Secretins form higher-ordered ring-like structures, which in the case of PulD are organized in the form of two rings connected by a central disc. This basic architecture creates two chambers of different size, one of which extends with its N-terminal domain into the periplasmic space as visualized by cryo electron microscopy of the trypsin resistant core of the PulD complex [21]. Comparison of the PulD structure with the needle complex shows striking similarities between the PulD rings and the outer rings of the base substructure of the needle complex. The similarities also extend to regions of the neck of the needle complex, which connect the outer rings with the inner rings (**Fig. 1A**). These similarities strongly suggest that InvG forms the outer rings of the needle complex of the SPI-1 T3SS base. However, this has not been formally demonstrated and the extent to which InvG may form the neck region of the needle complex has not been experimentally determined. In order to ascertain what needle complex substructures are specifically formed by InvG, we purified needle complexes from a *ΔinvG* mutant strain and analyzed their structure by cryo electron microscopy and single particle analysis (**Fig. 1**). Western blot analysis of the structures isolated from the *ΔinvG* mutant showed the presence of PrgH and PrgK at equivalent stoichiometry to that found in needle complexes isolated from wild type (**Fig. 1B**). Structures isolated from this mutant strain showed the presence of the inner ring 1 (IR1) and 2 (IR2) and an extending needle-like structure but lacked the upper rings and the neck region (**Fig. 1C, E**). Subtraction of two-dimensional class averages of samples obtained from a *ΔinvG* mutant strain (**Fig. 1E**) from a wild-type needle complex structure (**Fig. 1D**) revealed that InvG is localized at the apical side of the needle complex forming the outer ring structures and neck region (**Fig. 1F**). In comparison to PulD, InvG extends further into the periplasmic space reaching the largest inner ring (IR1) of the needle complex, presumably anchoring the outer rings and thus stabilizing the base substructure.

The secondary structural features of PrgH and PrgK strongly suggest that they form the inner rings of the needle complex base although no direct evidence for this hypothesis has been presented. Subtraction of two-dimensional class averages of samples obtained from a *ΔinvG* mutant strain from the wild-type needle complex structure also revealed that the ring structures from this mutant strain are virtually identical to the inner rings of the wild type needle complex (**Fig. 1F**). Since, beside PrgH and PrgK, the needle and inner rod proteins are the only main structural components of the *ΔinvG* substructure, these results formally demonstrate that PrgH and PrgK make up the inner rings of the base substructure. Taken together these results demonstrate that InvG is the main component of the outer rings and connecting neck of the needle complex while PrgH and PrgK are the main components of the inner rings.

### Localization and topology of PrgH within the base substructure of the needle complex

**Figure 2:**
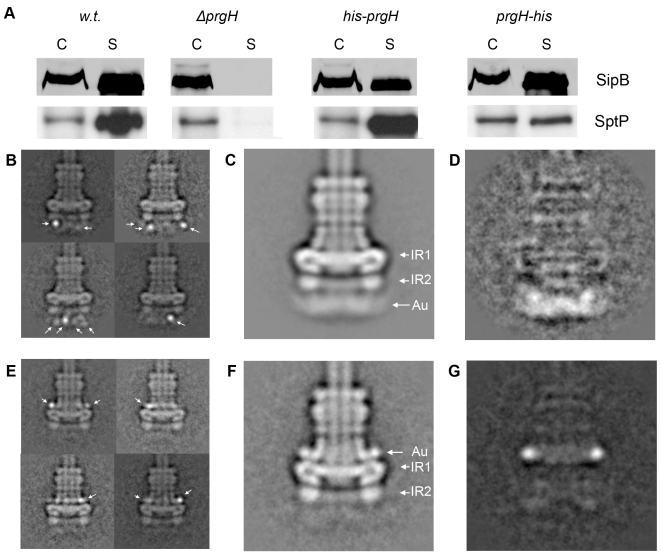
The N- and C-terminus of PrgH are located far away from each other within the needle complex. (**A**) N- and C-terminally poly-histidine tagged PrgHs are functional. Culture supernatants of a wild type *S. typhimurium* (w.t.), PrgH-deficient (*ΔprgH*), and mutant strains encoding either N- (his-PrgH) or C-terminally (PrgH-his) poly-histidine tagged PrgH were analyzed for the presence of the type III secreted proteins SipB and SptP by Western immunoblot. (C: whole cell lysates; S: culture supernatants). (**B**, **E**) Representative class averages obtained by single particle analysis of cryo electron microscopy images of Ni-NTA-labeled needle complexes derived from strains expressing either N-terminally (**B**) or C-terminally (**E**) tagged PrgH. (**C**, **F**) The total class averages (average of *all* particles) from the respective data set are shown in (**C**) (N-terminally labeled PrgH) and (**F**) (C-terminally labeled PrgH). The diffuse appearance of density at the basal side in (**C**) indicates that the Ni-NTA-nanogold (Au) label is present in various positions below IR2, but is more restricted above IR1 in C-terminally labeled complexes (**F**) (IR1 and IR2 = inner ring 1 and 2). (**D**, **G**) Density difference between the total averages of the labeled particles and unlabeled wild type complexes (panel D: w.t. needle complexes subtracted from labeled N-terminally tagged complexes (**2D** = **2C**-**1D**); panel G: w.t. needle complexes subtracted from labeled C-terminally tagged complexes (**2G** = **2F**-**1D**)).

Secondary structure prediction analysis indicates that PrgH contains a transmembrane domain (from amino acid 142 to 162), which would separate the protein into two soluble domains of roughly equivalent size but distinct secondary structures (**Fig. S1, S2**). The N-terminal domain is predicted to be rich in beta-sheets, whereas the recently solved crystal structure of the major part of the C-terminal domain (amino acids 170–362) showed that it has a modular arrangement of similar α/β domains [15]. The localization of the two soluble domains of PrgH relative to the assembled needle complex, however, is still unclear. This information is essential to guide the potential placement of the recently solved atomic structure of a soluble domain of PrgH within the protein density map of the needle complex. In order to determine the topology of PrgH within fully assembled complexes we constructed *S. typhimurium* strains expressing N- or C-terminal poly-histidine tagged PrgH. Addition of the peptide tag did not affect the functionality of the needle complex as assayed by its ability to secrete the effector proteins SipB and SptP (**Fig. 2A**). Needle complexes isolated from these strains were labeled with Ni-NTA nanogold and analyzed by cryo electron microscopy and single particle analysis. Class-averages showed that the labeled N- and C-termini are located far away from each other, consistent with the bioinformatic prediction that the different termini of PrgH are topologically located on opposite sides of the inner membrane (**Fig. 2B and 2E**). Classification of gold-labeled needle complexes isolated from a strain expressing N-terminal poly-histidine tagged PrgH showed additional density exclusively on the cytoplasmic side of the base substructure suggesting that the N-terminus of PrgH faces the cytoplasm (**Fig. 2B**). Moreover, in side views of classified particles, the nanogold label can be seen at various vertical and horizontal positions below IR2, presumably due to a flexible tag and/or the existence of multiple copies of PrgH that are organized in a cylindrical fashion and that are not uniformly labeled by Ni-NTA nanogold. This observation is consistent with the diffuse appearance of the additional “density” observed below IR2 in a class average of *all* the labeled particles (**Fig. 2C and D**). In contrast, labeled needle complexes isolated from a strain expressing C-terminal poly-histidine tagged PrgH showed additional density above IR1 in close proximity to the neck region (**Fig. 2E–G**). In this case, the labeling was visualized as a distinct density, even in the class average of all particles suggesting a more rigid conformation of this domain of PrgH. Taken together, these results indicate that the amino terminus of PrgH faces the bacterial cytoplasm while its carboxy terminus is located within the periplasm of the bacterial envelope.

### Localization of PrgK within the base substructure of the needle complex

**Figure 3:**
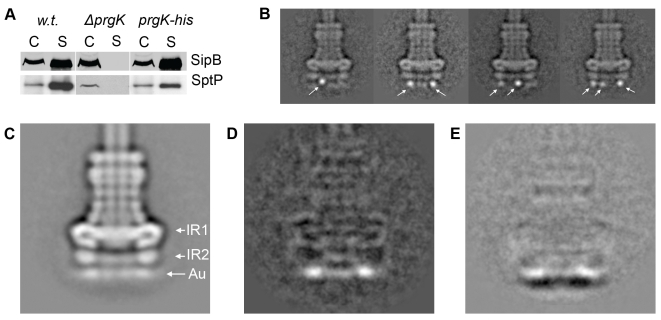
The C-terminus of PrgK is located at the basal (cytoplasmic) side of the needle complex. (**A**) Carboxy terminally poly-histidine tagged PrgK is functional. Culture supernatants of wild type *S. typhimurium* (w.t.), PrgK-deficient (*ΔprgK*), and a mutant strain encoding C-terminally (PrgK-his) poly-histidine tagged PrgK were analyzed for the presence of the type III secreted proteins SipB and SptP by Western immunoblot. (C: whole cell lysates; S: culture supernatants). (**B**) Representative class averages obtained by single particle analysis of cryo electron microscopy images of Ni-NTA-labeled needle complexes derived from strains expressing C-terminally poly-histidine tagged PrgK. (**C**) Total class average of Ni-NTA-labeled needle complexes obtained from a *S. typhimurium* strain expressing C-terminally poly-histidine tagged PrgK. The location of the density observed at the basal side (Au) is similar to the diffuse density observed in Ni-NTA-labeled needle complexes obtained from strains expressing N-terminally poly-histidine tagged PrgH (see **Fig. 2C**) (IR1 and IR2 = inner ring 1 and 2). (**D**) Resulting difference in density after subtraction of w.t. needle complexes from labeled C-terminally poly-histidine tagged PrgK complexes (**3D** = **3C**-**1D**). (**E**) Subtraction of the total averages of labeled complexes obtained from a strain expressing N-terminally-tagged PrgH from a strain expressing C-terminally-tagged PrgK (**3E** = **3D**-**2C**).

PrgK is a lipoprotein with a canonical *sec*-dependent transport signal sequence that is processed upon secretion [6], and a predicted single transmembrane domain (AA 207–227) close to its carboxy terminus. Consequently, the large N-terminal domain of PrgK (starting from Cys-18) is predicted to be localized in the periplasm and anchored to the inner membrane via its transmembrane domain. The atomic structure of the PrgK homologue EscJ, which lacks a transmembrane domain, revealed that this protein is organized in two independent domains linked by a flexible linker [13],[14] (**Fig. S1**). Although EscJ carries a ‘ring forming’ motif similar to that of PrgH and EscC (InvG) [15], EscJ rings have so far never been isolated and/or visualized. To investigate the topology of PrgK within the needle complex, we constructed *S. typhimurium* strains expressing N- or C-terminal poly-histidine tagged PrgK. Addition of the tag at the amino terminus resulted in a loss of type III dependent protein secretion (data not shown). In contrast, addition of the tag to the carboxy terminus did not negatively affect needle complex function as measured by SipB and SptP secretion (**Fig. 3A**). Needle complexes isolated from this strain, labeled with Ni-NTA and examined by cryo electron microscopy and single particle analysis showed additional density exclusively on the cytoplasmic side of the base substructure (**Fig. 3B**). These results indicate that the carboxy terminal domain of PrgK faces the cytoplasm. Comparison of the position of the nanogold label on the C-terminus of PrgK (**Fig. 3C**) with that of the N-terminus of PrgH (**Fig. 2C**) by subtracting the total class averages from wild type (**Fig. 1D**) showed a more distinct localization of the nanogold label in PrgK (**Fig. 3D** **,** **2D**). This observation suggests that the C-terminus of PrgK is less flexible than the N-terminus of PrgH and positioned closer towards the cup region of the needle complex (**Fig. 1A** **,** **3E**).

### Localization of PrgH and PrgK within IR1 and IR2

**Figure 4:**
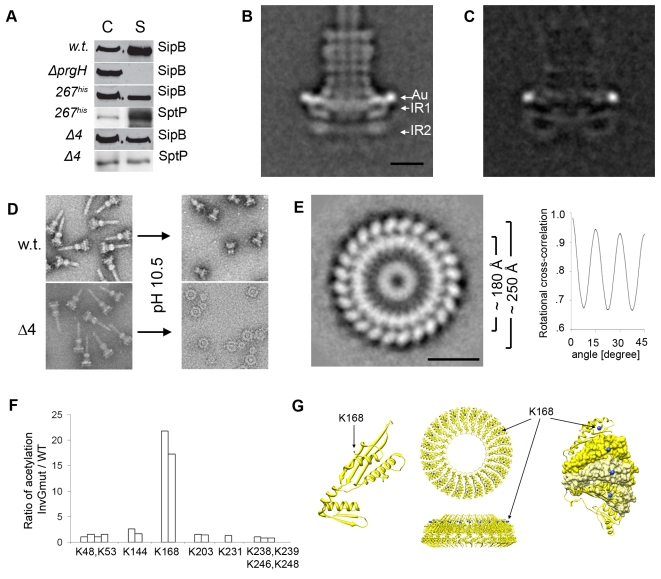
Organization of PrgH and PrgK within the lower ring of the needle complex. (**A**) Insertion of a poly-histidine linker at amino acid 267 of PrgH (267^his^) or removal of four amino acids from its C-terminus (Δ4) does not alter its function. Culture supernatants of wild type *S. typhimurium* (w.t.), PrgH-deficient (*ΔprgH*), and mutant strains encoding a poly-histidine linker at amino acid 267 of PrgH (267^his^) or a PrgH lacking the terminal four amino acids (Δ4) were analyzed for the presence of the type III secreted proteins SipB and SptP by Western immunoblot. (C: whole cell lysates; S: culture supernatants). (**B**) Representative class average obtained by single particle analysis of cryo electron microscopy image of Ni-NTA-labeled needle complexes obtained from a *S. typhimurium* strain expressing PrgH with a poly-histidine tag inserted at amino acid 267. A prominent gold label (Au) is seen at the widest side of the periplasmic face of the IR1 (IR1 and IR2 = inner ring 1 and 2). Bar = 10 nm. (Additional results of the analysis are shown in **Fig. S4**). (**C**) Resulting difference in density after subtraction of unlabeled w.t. needle complexes from labeled complexes isolated from strains expressing a poly-histidine insertion following amino acid 267 in PrgH. (**D**) Mutant needle complexes carrying truncated PrgH can be selectively disassembled into larger and smaller rings by shifting pH to 10.5 and subsequent negative staining, whereas wild type (w.t.) needle complexes maintain the integrity of the base. (**E**) *En face* class-average derived from single particle analysis from negatively stained electron microscopy images of inner rings substructures. The substructures were obtained by selective disassembly of needle complexes isolated from a mutant strain encoding for a C-terminally, four amino acid truncated PrgH. The ring substructure is organized in two larger concentric rings with different diameters (∼180Å and ∼250Å). Bar = 10nm. Rotational cross-correlation analysis revealed that the maximum of the cross-correlation peak is repeatedly obtained every 15°, demonstrating that the larger concentric rings of the inner ring structure from the *prgHΔ4* mutant strain exhibit 24 fold symmetry. (**F**) Surface accessibility of lysine K168 is increased in complexes obtained from a *S. typhimurium ΔinvG* strain. Primary amines of wild type and *ΔinvG* mutant complexes were acetylated with Sulfo-NHS-Acetate and the ratio of modified to non-modified peptides was determined by mass spectrometry. Each bar represents the ratio of acetylation of lysines between specific PrgK peptides obtained from the *ΔinvG* mutant and the same peptides obtained from a wild type complex (**Table S1**). The ratio of peptide acetylation is an average of two independent mass spectrometry measurements. (**G**) Lysine 168 is surface exposed in modeled PrgK rings. A monomeric PrgK structure was modeled using EscJ as a template and protein-protein contacts present in the EscJ crystal structure were used to form a PrgK ring (top and side view). Lysine 168 is located on top of the ring and its side group nitrogen is surface exposed as highlighted in blue in the surface view of the cut-out segments of individual monomers. The cut-out segment shows two PrgK molecules in surface representation (yellow and light-yellow) followed by one neighboring molecule on each side but displayed in ribbon style.

In order to refine the relative position of PrgH within the inner rings we constructed a strain of *S. typhimurium* that expresses a PrgH mutant in which we introduced a poly-histidine insertion linker after amino acid 267 (PrgH-267^his^) (**Fig. S3**). The resulting strain expressed a functional T3SS system as shown by its ability to secrete the effector proteins SipB and SptP (**Fig. 4A**). Needle complexes isolated from this strain were labeled with Ni-NTA-nanogold and examined by cryo electron microscopy and single particle analysis. Additional density at the widest part of the IR1 (**Fig. 4B, 4C** **, Fig. S4**) was readily observed, indicating that PrgH is located at the periphery of IR1.

PrgK has been proposed to exist as an inner ring enclosed by an outer ring formed by PrgH [15]. This model was based on the crystallographic analysis of the PrgK homolog EscJ, which crystallized in a superhelical fashion. Subsequent modeling led to an approximately 180Å wide ring structure, which was proposed to be anchored to the inner membrane by the lipidated N-terminal cysteine of EscJ/PrgK. In this model, the EscJ/PrgK ring would be located on top of the outer leaflet of the inner membrane projecting into the periplasmic space [14]. An alternative model proposed for MxiJ, the Shigella spp. homolog of PrgK, positions this protein as radial spikes enclosed by an outer ring [16]. To gain insight into the organization of PrgK within the needle complex, we sought to obtain top views of the inner rings of the needle complex by electron microscopy for subsequent single particle analysis, which required the removal of the InvG rings. The proximity of the C-terminus of PrgH to InvG suggested the possibility that truncating the C-terminus of PrgH could weaken the interaction between these two proteins. We therefore constructed a S. *typhimurium* strain that expresses a PrgH mutant lacking its last 4 amino acids (PrgH^Δ4^). The resulting strain expressed a functional T3SS as shown by its ability to secrete effector proteins (**Fig. 4A**) and the presence of intact needle complexes (**Fig. 4D**). However, when subjected to high pH treatment, the needle complexes not only disassembled the needle filament over time [9], but, in contrast to wild type, upon negative staining the complexes obtained from this mutant strain could be further disassembled to generate intact inner rings (IR1/IR2) separated from the smaller outer rings (**Fig. 4D**). We analyzed the inner rings (IR1/IR2) thus obtained by electron microscopy without imposing any symmetry or introducing any potential model bias. This analysis showed the presence of several concentric rings with different staining intensities (**Fig. 4E**). The two concentric rings with the larger diameter (∼250Å and ∼180Å) exhibited repeating subunits separated every 15 degrees thus resulting in a 24-fold symmetry. The accessibility of PrgH-267^his^ to surface labeling and the appearance of additional density on the outermost surface of IR1 strongly suggested that the largest ring visualized in the inner ring particles must be made up by PrgH. On the other hand, the size, configuration, and density distribution of the second largest ring with an approximate diameter of 180Å are consistent with the hypothesis that this ring is formed by PrgK (**Fig. 4E, G** **, Fig. S6**). If this were the case, most of PrgK would be buried by the presence of InvG (top) and PrgH (outside). Consequently, this model would also predict that removal of InvG should expose otherwise buried residues. To test this hypothesis, we compared the ratio of accessibility of lysine residues of PrgK in complexes isolated from wild type or *ΔinvG S. typhimurium* strains (which exposes the apical side of IR1, see **Fig. 1**) by subjecting the isolated particles to acetylation using NHS-acetate and subsequent analysis by mass spectrometry. Lysine 168 from PrgK was found to be more frequently (>15 times) acetylated in tryptic peptides derived from PrgK obtained from complexes from a *ΔinvG* mutant strain than in PrgK peptides obtained from wild type needle complexes (**Fig. 4F** **, Table S1**). Consistent with this observation, modeling of PrgK using the EscJ structure as a template (see below) showed that the side chain amino group of K168 would be surface exposed in the absence of InvG (**Fig. 4G**).

Taken together these results indicate that the inner rings are composed of two larger concentric rings: a peripheral ring formed by PrgH enveloping an inner ring formed by PrgK, which is shielded by PrgH (on the sides) and InvG (on the top).

### Domain interactions among components of the type III secretion needle complex characterized by cross-linking and mass spectrometry

**Figure 5:**
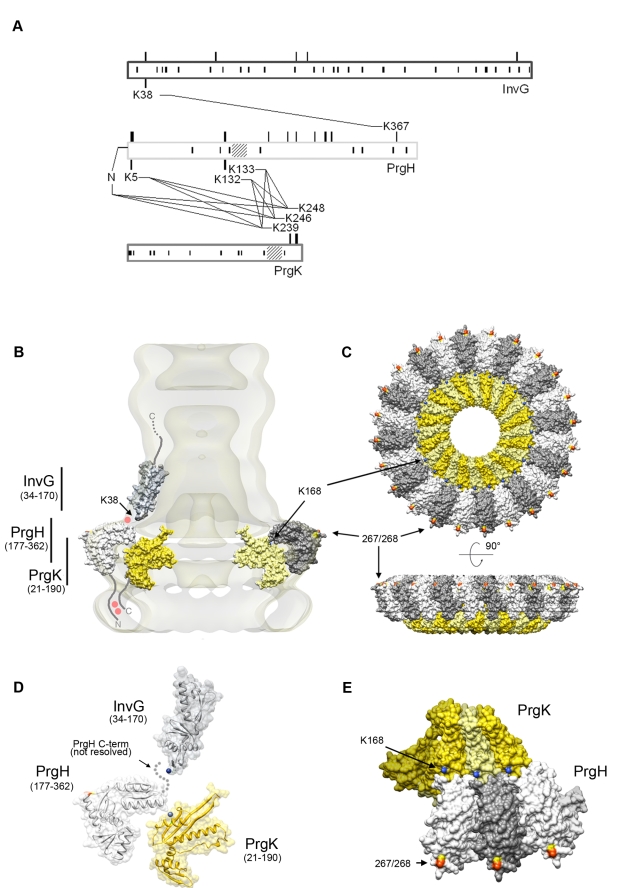
Domain interactions and relative orientations of needle complex components. (**A**) Proximity of specific domains of the base proteins, InvG, PrgH, and PrgK within the needle complex. The block diagrams shows the three major base proteins, InvG, PrgH, and PrgK and covalent cross-links of peptides obtained from chemically derivatized needle complexes at primary amino groups. Amino acid position are indicated for the full length proteins prior signal peptide cleavage (processed InvG starts at Ser-25, and processed PrgK starts at Cys-18 [6]) protein-cross-links found are indicated with amino acid position and with crossing lines between proteins. While the position of non-derivatized lysines (presumably due to lack of surface exposure) is shown as vertical lines *within* the block diagram, the positions of derivatized lysines (surface accessible) is indicated as vertical lines extending from the block diagram (**Table S2**). Note that lysines within the N-terminal domain of PrgK are not derivatized, suggesting that the majority of PrgK within fully assembled needle complexes is not surface exposed. (**B–E**) Topographic model of the needle complex: Localization of InvG, PrgH, and PrgH within the base of the needle complex. The N-terminal domain of InvG (blue-grey) reaches far down into the neck region and is in close contact with the C-terminal domain of PrgH (white and grey), which resembles the larger of the two concentric rings. Insertion of a poly-histidine tag between amino acid 267 (yellow) and 268 (orange) and subsequent Ni-NTA nanogold labeling further determines the position of this domain within the complex. Sites of interaction found by cross-linking and mass spectrometry, for which in the case of PrgH and EscJ/PrgK no atomic structure is available, are labeled as red dots. The N-terminal domain of PrgH is pointing to the cytoplasmic side of the complex, and interacts with the C-terminal domain of PrgK. For both, no high resolution structure is available as of yet. The N-terminal domain of PrgK is located within the complex and is therefore packed into its position by PrgH from the side and InvG from the top. (**C**) Top and side view of the modeled PrgH (white/grey alternating) and PrgK (yellow/bright yellow alternating), as well as sites accessible for nanogold labeling (267/268) and chemical derivatization (K168). (**D**) Proposed relative position of protein domains from PrgH, InvG, and PrgK. The side group nitrogen of K38 (InvG) and K168 (PrgK) are highlighted in blue. (**E**) Top-view of three PrgK and PrgH monomers extracted from the modeled inner ring structure highlighting sites for chemical derivatization (PrgK (K168, blue)) and Ni-NTA nanogold labeling of a poly-histidine insertion at position 267/268 (yellow/orange) within PrgH.

In order to refine the spatial relationship between the domains of the different proteins that make up the needle complex and to gain insight into the nature of potential domain-domain interactions, we used chemical cross-linking combined with high-resolution mass spectrometry. Purified needle complexes were incubated with the bi-functional cross-linking agent BS2G (d0/d4), which is able to covalently link primary amino groups at a distance up to ∼8Å. Particles in which two or more complexes were cross-linked were separated from individual ones by re-purification on sucrose gradients, and tryptic fragments generated from these complexes were analyzed by mass spectrometry after protease digestion in solution. Several cross-links were identified between the different domains of InvG, PrgH, and PrgK (**Fig. 5A** **,** **Table 1**). A single cross-link between InvG (K38) and PrgH (K367) was found, establishing that the N-terminal domain of InvG and the C-terminal domain of PrgH are in close contact. This is also consistent with the observation that truncation of four amino acids from the carboxy terminal end of PrgH resulted in the destabilization of the needle complex (**Fig. 4D**) and supports the notion that the interaction of PrgH with InvG is very important for the linkage of these two substructures. From strains with longer truncations at the C-terminus of PrgH only complexes similar to those obtained from a *ΔinvG* mutant strain could be purified (data not shown), further demonstrating the importance of this domain for needle complex assembly.

**Table 1:** List of hetero-cross-linked peptides determined by chemical derivatization and mass spectrometry.

Crosslinked proteins	Crosslinked lysines	Peptide1	Peptide2	Mass Error (ppm)	xQuest Score
**InvG-PrgH**	K38—K367	IPVTGSGFVA**K**DDSLR	DDWL**K**G	2.1	17.7
**PrgH-PrgK**	N-Term-K239	**M**ETSK	**K**GITADDK	1.3	25.8
**PrgH-PrgK**	N-Term-K246	**M**ETSK	GITADD**K**AK	1.3	26.7
**PrgH-PrgK**	N-K248	**M**ETSK	KGITADD**K**AK	0.7	19.1
**PrgH-PrgK**	N-K248	**M**ETSK	A**K**SSNE	1.1	20.5
**PrgH-PrgK**	K5-K239	METS**K**EK	**K**GITADDK	0.5	21.4
**PrgH-PrgK**	K5-K246	METS**K**EK	GITADD**K**AK	1.0	18.4
**PrgH-PrgK**	K5-K248	METS**K**EK	A**K**SSNE	0.0	15.4
**PrgH-PrgK**	K132-K239	LETSA**K**K	**K**GITADDK	1.4	23.4
**PrgH-PrgK**	K132-K246	LETSA**K**K	GITADD**K**AK	0.9	15.2
**PrgH-PrgK**	K132-K248	LETSA**K**K	A**K**SSNE	1.0	22.4
**PrgH-PrgK**	K133-K239	**K**NEPR	**K**GITADDK	1.1	21.2
**PrgH-PrgK**	K133-K246	**K**NEPR	GITADD**K**AK	1.3	24.7
**PrgH-PrgK**	K133-K248	**K**NEPR	A**K**SSNE	1.7	17.8

Only peptides with an xQuest score of higher than 15 are displayed as those cross-linking results are likely to be reliable [25] (Text S1). The respective modified amino acid is displayed in bold. Under “Crosslink” listed positions are the absolute positions of the respective protein (including signal peptides), “Error” the deviation of measured precursor ion mass from theoretical precursor ion mass in parts per million, and “Score” the xQuest score.

We found two clusters of residues of the amino terminus of PrgH that cross-linked to the C-terminal end of PrgK (**Table 1** and **Fig. 5A**). In addition to the primary amino group at the N-terminal end of the protein, lysine residues at position 5, 132, and 133 of PrgH were found to cross-link to lysine residues at position 239, 246 and 248 of PrgK. The linkages found exclusively between the N-terminal domain of PrgH and the C-terminal tail of PrgK are in full agreement with the results obtained from the nanogold labeling experiments. The absence of any cross-links from the large N-terminal domain of PrgK is also consistent with the proposed concentric arrangement of PrgK shielded by PrgH and InvG.

### Topology model of the *S. typhimurium* needle complex components

To construct a model for the topological arrangement of the components of the *S. typhimurium* needle complex, we used the available crystal structure of PrgH and modeled InvG and PrgK using the structures of their close homologues EscC and EscJ, respectively (Text S1). In addition, we incorporated in such model all of our experimental findings described above. The model shows that the N-terminal domain of PrgH and the short C-terminal domain of PrgK are in close contact at the cytoplasmic face of the needle complex (**Fig. 5B**). Furthermore, the N-terminal domain of InvG is localized at the lowest part of the neck-region oriented in such a way that allows its interaction with the C-terminal domain of PrgH (**Fig. 5B** **, Fig. S7**). This arrangement is supported by the finding of cross-linked peptides [IPVTGSGFAV**K_38_**DDSLR]-[DDWL**K_367_**G] encompassing these two domains (**Table 1**). The C-terminal PrgH peptide, however, is not visible in the atomic structure, hence this interaction can not be incorporated into a high-resolution model building (**Fig. 5D**).

Several models involving 12 or 14 fold symmetries have been proposed for the N-terminal region of the InvG homologue EscC. These ring models were placed at different positions within the needle complex depending on the proposed symmetries and correlating different volumes and diameters [15]. Our data indicate that the amino terminus of InvG should face the inner rings, which provides an unambiguous orientation to the InvG ring that is incompatible with some of the previously proposed models [15],[16]. However, our data cannot clarify the issue of the differing symmetries in the proposed models, which would require experimental data at a sufficiently high resolution to show individual subunits within the outer ring and neck region.

PrgH and PrgK are the main constituents of the inner rings. Nanogold labeling of a needle complex isolated from strain expressing a PrgH mutant derivative with a his-tag after position 267 resulted in an additional density on the outer perimeter of the IR1. This observation led us to manually position the C-terminal domain of PrgH in such a way that amino acid 267 would be located at the periphery of IR1 (**Fig. 5C, E** **, Fig. S7**). The positioning of PrgK in our model takes into account the crystallographic contacts between the monomers observed in its homologue EscJ, resulting in a ring of roughly the same diameter to that proposed for EscJ. In our model we have also positioned the PrgK ring in such a way that PrgK K168 faces the periplasm, to account for our observation that K168 becomes accessible for derivatization in the absence of InvG (**Fig. 5C, E** **, Fig. S7**).

## Discussion

The atomic structures of several soluble domains of needle complex components have become available. Attempts have been made to place those domains within the available structures of the entire needle complex to begin to generate an atomic model of the entire structure. However, the low resolution of the available needle complex structure has significantly hampered this objective and has prevented the unambiguous assignment of specific domains to specific protein densities within the needle complex. The cylindrical architecture of the needle complex further complicates the docking since, depending on the assumed stoichiometry or subunit number, modeled ring-like structures can be placed at different positions within the needle complex. Consequently, different studies have proposed incompatible locations for different proteins and/or protein domains within the needle complex [15],[16]. We have used a multi-pronged approach to generate data allowing us to place domains of different protein components of the needle complex at specific sites of its structure. We have experimentally shown that InvG forms the outer rings. Although widely predicted from secondary structure analysis as well as the organization of homologues in other secretion systems (e.g. PulD family of proteins), this is the first experimental demonstration of this organization. Furthermore, our data demonstrated that InvG reaches deep into the periplasmic space making up the entire neck region and making direct contact with the inner rings, and in particular, the carboxy terminal domain of PrgH. Although the C-terminus of PrgH is not visible in the atomic structure therefore hindering high-resolution model building, the interaction between this domain and the amino terminus of InvG appears to be critical for the stability of the entire complex.

Our data also demonstrates that PrgH and PrgK are the main constituents of the inner rings. Our results indicate that the C-terminal domain of PrgH is localized at the periphery of IR1, as supported by the nanogold label of a tag introduced between amino acid 267 and 268, **Fig. 4B and 4C**). As previously proposed [15], our results suggest that PrgK is organized in a smaller diameter ring structure engulfed by the PrgH ring on the side and covered by the InvG neck region on top.

Multicomponent macromolecular complexes are central to many fundamental processes in biology. To gain insight into mechanistic details, knowledge not only of the atomic structure of the different subunits, but also their orientation relative to one another is essential. Therefore, our studies offer an essential view of the architecture of this remarkable bacterial nanomachine and will be the basis for further functional studies.

## Materials and Methods

### Bacterial strains and plasmids

All strains were derived from the non-flagellated *S. enterica* serovar *typhimurium* strain SJW2941. The *invG*, *prgH*, and *prgK* mutant alleles were introduced into this strain by P22HT*int*-mediated transduction as described elsewhere [22]. The needle complexes lacking InvG were purified from the strain SB1171 (all “SB” strains are described elsewhere) [10]. PrgH modified needle complexes were obtained by complementing the strain SB906, which harbors a chromosomal deletion of *prgH*, with plasmids expressing C-terminal truncated PrgH, an 18x-C-terminal, a 6x-N-terminal, or a 6x internal (after M267) poly-histidine tagged PrgH. A longer C-terminal poly-histidine tag was chosen because a 6xhistidine-tag yielded a poor Ni-NTA-NanoGold label. All plasmids were based on the low copy vector plasmid pWSK29 and expressed the tagged PrgH under its natural promoter (500bp upstream sequence). The w.t. strain SB905 was used as a negative control for the Ni-NTA-NanoGold labeling experiments and for the detection of adjacent epitopes by mass spectrometry. An allele of PrgK with a C-terminal 6x-poly-hisitidine tag was introduced into SB905 by homologous recombination as described previously [22]. Plasmids expressing the *hilA* positive transcriptional regulator gene under the control of *P*_BAD_ promoter (pSB667 or pSB1418) were used to over-express the SPI-I TTSS regulon as described elsewhere [23].

### Bacterial secretion assay

The preparation and analysis of cultured supernatant proteins was conducted as described elsewhere [22].

### Needle complex expression and purification

Needle complex purification was based on the purification protocol previously published [9]. Needle complexes were purified from 2 L of bacterial culture. For the strain SB1171 the protocol was up-scaled to 18 L of bacterial culture to obtain a yield comparable to the other strains. Details are provided under Text S1. To further improve purity of the sample or to separate inter-particularly cross-linked particles from single particles, a sucrose gradient centrifugation was performed. A continuous 10–25% sucrose gradient in thin-wall tubes of the Sorvall TH-660 rotor was made with a 0.1% LDAO, 10 mM sodium phosphate (pH 7.4), 0.5 M NaCl buffer. The sample was applied to the top of the gradient and centrifuged for 3.5 hours at 50 krpm. Two 1.1 ml and four 0.45 ml fractions were collected from the top of the tube and were diluted in sucrose-free buffer and pelleted at 90 krpm for 30 minutes in a Sorvall S100-AT4 rotor. The pellets were re-suspended in 0.1 ml 0.1% LDAO, 10 mM sodium phosphate (pH 7.4), 0.5 M NaCl. Integrity and purity of the sample was verified by negative stain EM and SDS-PAGE.

### Disassembly of needle complexes to inner and outer rings

The needle filament was removed by incubating purified needle complexes in 10 mM sodium phosphate (pH 10.4), 0.5 M NaCl, 0.1% LDAO for 15 min at 37°C. Subsequently the pH was re-adjusted by purifying the sample by sucrose gradient centrifugation as described above. The sample was observed by negative stain electron microscopy as described below.

### Ni-NTA-NanoGold labeling of poly-histidine-tagged needle complexes

Samples tending to aggregate (His-PrgH, PrgK-His) were purified in presence of 10mM imidazol. Free thiols of sucrose gradient purified needle complexes were blocked by incubating the sample with 1 mM N-ethylmaleimide at 4°C over night. Subsequently, imidazole was added to a concentration of 20 mM and the sample was incubated with 5 µM nickel-nitrilotriacetic acid (Ni-NTA) NanoGold (Nanoprobes, Stony Brook, NY) at room temperature for 10 minutes. The sample was gel-filtrated with a Sephacryl 300 column to remove unbound Ni-NTA-NanoGold and analyzed by cryo-electron microscopy.

### Chemical derivatization, mass spectrometry (MS) and MS data analysis

Chemical derivatization was performed by incubating sucrose gradient purified sample (about 1mg/ml) on ice with 600 µM Sulfo-NHS Acetate (Pierce, Rockford, USA) for 60 minutes or 200 µM BS2G-d0/d4 (Pierce, Rockford, USA) for either 30 or 60 minutes.. Details about chemical derivatization and subsequent MS analysis are provided in Text S1.

### Electron microscopy and image processing

Samples were applied to glow-discharged carbon-coated 400 mesh hexagonal Cu/Pd-grids. For negative stain images 5 µl of sample was applied to the grid and subsequently stained with 2% PTA (phosphotungstate), pH 7.0 (**Fig. 4D**) or NanoVan (Nanoprobes, Stony Brook, NY) (**Fig. 4E** **, Fig. S5**). Overview images were acquired at 44,000-fold magnification in a Morgani TEM (FEI Company, Hillsboro, USA) at 80kV using an 11 megapixel CCD camera. High-resolution data was collected with a FEI Tecnai Polara at 300kV using a Gatan Ultrascan 4000 UHS CCD camera (16 mega-pixel, 4k×4k, 15 micron pixel size). Images were acquired at 112,968-fold magnification, which corresponds to 1.33 Å/pixel at the level of the specimen, with underfocus values ranging from 1.2–3.5 µm.

For cryo electron microscopy 5 µl sample was applied to glow-discharged grids before vitrification by plunge freezing in liquid ethane. Low-dose data was collected with a FEI Tecnai Polara at 300kV using a Gatan Ultrascan 4000 UHS CCD camera (16 mega-pixel, 4k×4k, 15 micron pixel size). Images were acquired at 71,949-fold magnification (2.08 Å/pixel) with under focus values ranging from 1.2–3.5 µm.

Individual particle projections were extracted, combined into a dataset and processed by IMAGIC-5 (Image Science Software GmbH, Germany). The contrast reversals imposed by the contrast transfer function (CTF) of the objective lens were corrected for each particle projection using the mean under focus value of the respective CCD-images as determined by the program CTFFIND3 [24].

## Supporting Information

Text S1 Supplementary Information Protocols
(0.05 MB RTF)

Figure S1 Block diagram of the three major base proteins, InvG, PrgH, and PrgK of the needle complex and region of atomic structures of PrgH and the homologues EscC (InvG) and EscJ solved. Vertical lines extending from the block diagram are positions of lysines that can be chemically derivatized and are presumably surface exposed. Remaining non-derivatizable (and probably not surface exposed) lysines are indicated as vertical lines within the block diagram.. Amino acid position for InvG, PrgH, and PrgK are indicated for the full length proteins prior signal peptide cleavage (processed mature InvG starts at Ser-25, and PrgK starts at Cys-18, respectively).
(2.84 MB TIF)

Figure S2 Sequence alignment and secondary structure prediction of the major part of the N-terminal domain of PrgH (1–122) from various species (S. typhimurium (PrgH), E. coli (EprH), Y. enterocolitica (Ye3550), S. flexneri (MxiG)). The N-terminal domain of PrgH is predicted to be mostly composed of beta-strands. Highly conserved and similar residues are indicated in red.
(0.47 MB TIF)

Figure S3 Ribbon diagram of PrgH (177–362) and position for insertion of a poly-histidine tag following amino acid 267 for Ni-NTA-nanogold labeling.
(0.23 MB TIF)

Figure S4 Single particle analysis of Ni-NTA labeled needle complexes with a poly-histidine insertion following position 267 in PrgH. Representation of various views of class-averages, variance-images, and individual particles of PrgH-267^his^ labeled needle complexes show the presence of the nanogold label at the outermost perimeter of the inner rings. The highest variance was observed at the position of the nanogold label, which indicates that the individual particles are not uniformly labeled. This is also evident, when individual particles are visualized.
(4.35 MB TIF)

Figure S5 PrgK resembles the smaller concentric ring of the PrgHΔ4 inner ring substructure. *En face* view of the most prominent class-average derived after hierarchical clustering from images of negatively stained inner rings substructures from PrgHΔ4 complexes. The smaller concentric ring shares similarity in dimension (∼180Å) and organization to the modeled PrgK ring. In order to allow a comparison between the class average of the inner ring substructure and the modeled PrgK ring, only a segment of the latter is shown.
(0.53 MB TIF)

Figure S6 Ribbon diagram of modeled InvG and PrgK domains. The structure of InvG (white) and PrgK (yellow) were obtained by structure homology-modeling based on EscC (blue) and EscJ (blue) templates, using the SWISS-MODEL server (http://swissmodel.expasy.org/). Note that in EscJ Asn134 to Gln139 is not resolved in the X-ray structure, however, the corresponding amino acids in PrgK (Asp133 to Lys144) have been modeled using the the SWISS-MODEL server, indicating a possible conformation of this amino acid stretch (marked with an *). This domain (also marked with an *) is shown with a 70% transparency setting in Fig. S7.
(0.43 MB TIF)

Figure S7 Overview of needle complex substructures and organization. Side view of half-sectioned base and location of individual protein domain. (* indicates to a possible confirmation of amino acids 133–144 in PrgK based on modeling using the SWISS-MODEL server (Fig. S6). Concentric ring-organization of IR1/2 revealed from top-viewed disassembled complexes and ring models of PrgH and PrgK.
(1.48 MB TIF)

Table S1 Comparison of surface accessibility in wild type and Δ*invG* mutant complexes. Peptides from NHS-acetate derivatized complexes were identified by MS/MS sequencing and semi-quantified by MS peak integration (Text S1). The table displays the modified lysine (Lysine), the identified corresponding peptide (Peptide) and the degree of NHS-acetate modification for the wild type (%w.t.) and the *ΔinvG* mutant (%ΔinvG). The ratio of peptide acetylation is an average of two independent mass spectrometry measurements. To highlight which lysines get stronger modified in the *ΔinvG* mutant (and are most likely more surface exposed) the ratio of *ΔinvG* mutant and wild type acetylation is determined (%ΔinvG/%w.t.). Due to the presence of several lysines within the peptides “K48, K53” and the C-terminally located peptides (“C-term” = K238, K239, K246, K248) the exact position of the derivatization could not be distinguished.
(0.07 MB RTF)

Table S2 List of mono-links and loop-links determined by chemical derivatization and mass spectrometry. In order to specify all lysines being accessible for derivatization, hetero-cross-links (cross-link between two different proteins, see Table 1), mono-links (modified peptides that reacted with only one functional group of the bivalent cross-linker (BS2G (0/d4))) and loop-links (cross-links within one polypeptide chain or between homo-proteins) were determined. Only peptides with an xQuest score of higher than 15 are displayed as those cross-linking results are likely to be reliable (see Text S1). The respective modified amino acid is displayed in bold. Under “Monolink/Looplink” listed positions are the absolute positions of the respective protein (including signal peptides), “Error” the deviation of measured precursor ion mass from theoretical precursor ion mass in parts per million, and “Score” the xQuest score.
(0.10 MB RTF)
