# Supplementary material for: Topology and Organization of the Salmonella typhimurium Type III Secretion Needle Complex Components
Source: PLoS Pathog. 2010 Apr 1;6(4):e1000824. doi: 10.1371/journal.ppat.1000824 (PMC2848554; doi:10.1371/journal.ppat.1000824)
Supplement: Text S1 — Supplementary Information Protocols (0.05 MB RTF) [file ppat.1000824.s001.rtf]

SUPPLEMENTARY INFORMATION

Title:
Topology and organization of the Salmonella typhimurium type III secretion needle complex components

Authors:
Oliver Schraidt1,2, Matthew D. Lefebre3, Matthias J Brunner1,2, Wolfgang H Schmied1,2, Andreas Schmidt4, Julia Radics1,2, Karl Mechtler1, Jorge E Galan3, and Thomas C Marlovits1,2

1Research Institute of Molecular Pathology, Vienna, Austria
2Institute of Molecular Biotechnology GmbH, Austrian Academy of Sciences, Vienna, Austria
3Yale University School of Medicine, Section of Microbial Pathogenesis, Boyer Center for Molecular Medicine, New Haven, CT , United States of America
4CD-Laboratory for Proteome Analysis, Vienna, Austria


Keywords:
Type III secretion, topology, cryo electron microscopy, cross-linking, mass spectrometry

Correspondence should be addressed to T.C.M email: marlovits@imp.ac.at


Supplementary Protocol

Needle complex expression and purification
Seed cultures were grown overnight at 37°C in L-broth supplemented with 0.3 M NaCl and appropriate antibiotics.  Cultures were diluted 1:10 in the same medium and grown to an OD of 0.5 before inducing expression of hilA by adding arabinose to a concentration of 0.012%.  During purification, samples were kept at 4°C unless stated otherwise.  Bacteria were harvested at early stationary phase, re-suspended in 150 mM sodium phosphate (pH 7.4), 0.5 M sucrose, 1.4 mg/ml hen egg lysozyme, 12.2 mM EDTA, and incubated on ice while stirring for 45 minutes followed by 15 minutes at 37°C.  Cells were lysed with 0.35% lauryldimethylamine oxide (LDAO) before adding 500 mM NaCl and 20 mM MgCl2 to the lysate.  Cell debris was removed by low-speed centrifugation in a Beckmann JLA-16.250 rotor at 13,900 rpm for 20 minutes and needle complexes were pelleted by high-speed centrifugation in Sorvall T-647.5 rotor at 40 krpm for 150 minutes.  The pellet was re-suspended in a 0.5% LDAO, 10 mM sodium phosphate (pH 7.4), 0.5 M NaCl, 5 mM EDTA buffer and adjusted to a final concentration of 27.5% w/v of CsCl.  Samples were centrifuged for 12 hours at 50 krpm in a Sorvall TH-660 rotor.  Half-milliliter aliquots were combined with 2.4 ml CsCl-free buffer and pelleted in a Sorvall S100-AT4 rotor at 90 krpm for 30 minutes.  The needle complexes were re-suspended in 0.1 ml 0.1% LDAO, 10 mM sodium phosphate (pH 7.4), 0.5 M NaCl.  

Chemical derivatization and sample preparation for MS
The cross-linkers BS2G-d0 and BS2G-d4 (Pierce, Rockford, USA) were dissolved in water-free DMSO to a concentration of 10 mM and combined at a molar ratio of 1:1.  The chemical derivatization agent Sulfo-NHS Acetate (Pierce, Rockford, USA) was dissolved in water to a final concentration of 10 mM.  Samples with a protein concentration of about 1 mg/ml were incubated on ice with 600 µM Sulfo-NHS Acetate for one hour or with 200 µM BS2G-d0/4 for half an hour or one hour.  Mass spectrometry data obtained from the 30-minute and the 60-min incubation was pooled.  The reaction was quenched with an excess of Tris-HCl.  The cross-linked sample was then subjected to sucrose gradient centrifugation to remove aggregates.  An aliquot of sample was analyzed by SDS-PAGE and western-blot with a polyclonal α-NC antibody.  The degree of cross-linking was estimated by comparing the density of shifted bands.
The chemically derivatized samples were reduced with 0.1 mM DTT at 56°C for 40 min and subsequently alkylated in the dark at room temperature by addition of 0.25 mM iodoacetamide for 30 min.  The reaction was quenched by 0.5 mM DTT.  Samples were digested with 1 μg trypsin (mass spectrometry grade; Promega, Madison, USA) per 15 µg sample at 37°C for 16 hours.  An additional 1 μg of trypsin was added and the sample was incubated for another 16 hours at 37°C.  Digestion was stopped by addition of 10% trifluoroacetic acid to a final concentration of 0.5%.  In case of acetyl-labeling, protein samples were cleaved by chymotrypsin, as unlabeled lysine in contrast to labeled ones a trypsin substrate, thus hampering a quantitative comparison between peptides.  

Mass spectrometry (MS)
After enzymatic digestion the peptides were separated by reversed-phase chromatography with a three-solvent gradient1 on an Ultimate 3000 DualGradient HPLC system (Dionex, Idstein, DEU) equipped with a C18 separation (15 cm x 75 ìm x 3 ìm) and a C18 trap column (5 mm x 200 ìm x 3 ìm; both Dionex, Acclaim, 100 Å).  Solvent A contained 5% acetonitrile, 0.1% formic acid (v/v), solvent B contained 30% acetonitrile, 0.1% formic acid (v/v), and solvent C contained 80% acetonitrile, 5% trifluoroethanol, 0.08% formic acid (v/v).  
Analytes were first separated for 190 minutes through a gradient from 100% A to 100% B before the solvent was switched to 60% B and 40% C within 2 minutes.  A second gradient (60% B, 40% C to 0% B, 90% C, 10% water) eluted stronger binding analytes in 60 minutes.  It was followed by a high organic wash and the reconstitution of the column.  A nano-electrospray ionization source directly connected the column outlet with the mass spectrometer.  MS and MS/MS analysis of the peptides was performed by a linear trap quadrupole Orbitrap mass spectrometer (Thermo Fisher Scientific, Waltham, USA) that operated in positive ionisation mode.  The applied duty cycle consisted of a MS survey scan, in which precursor ions were determined, and six MS/MS scans, of which three were acquired in the ion trap part of the instrument in collision-induced dissociation (CID) mode and three in the orbitrap in higher-energy C-trap dissociation (HCD) mode.  For each of the three selected precursors a CID and an HCD spectrum was acquired.  To identify chymotryptic peptides from acetyl labeling experiments during MS/MS data analysis the instrument applied CID fragmentation in case of doubly charged ions and electron transfer dissociation for fragmentation of three times or higher charged peptide ions.  

MS data analysis
Approximately 15000 to 20000 MS and MS/MS spectra of a single run were recorded into RAW files (with average size of more than 1GB), a proprietary binary format for MS data (Thermo Fisher Scientific, Waltham, USA).  MS peak data was deconvoluted and exported by the Xtract plug-in of the Qual-Browser to multiple files (Thermo Fisher Scientific, Waltham, USA).  Settings for mass extraction of monoisotopic masses from survey scans were a signal/noise cutoff of 5, maximum charge 10, Fit factor of 60% and maximum remainder of 20%.  The multiple files were converted into text format and combined to a list.  Isotopically labeled cross-linkers had been employed to produce mass doublets with a characteristic mass offset of 4.0247 Da between the light and the heavy isotopes.
Since no practical solution to find such characteristic doublets in datasets of this magnitude was available, we developed a software tool to fulfill this task.  Masses were paired into doublets according to their offset (ΔMr = 4.0247 Da, with a maximum deviation of 5 ppm), provided that both the heavy and the light mass were detected within 50 scans.  Similar peak intensity or peak area of the two peaks forming a doublet would have served as another useful criterion.  However, the data required to perform this analysis was not offered by the Xtract plug-in.  Usually about 90% to 99% of all masses could be discarded as irrelevant, as they could not be assigned to a specific doublet.  In order to further reduce the complexity of the dataset, the remaining masses were matched against a list of hypothetical cross-link masses between NC subunits, generated by in-silico digest of the protein sequences and exhaustive combination of the generated peptides.  This step further reduced the dataset complexity by a factor of three to five and left only several hundred cross-link candidates.
In order to confirm the cross-link candidates, their fragmentation spectrum was scored by the xQuest algorithm2.  The MS/MS spectra assigned to the putative cross-link precursors were submitted to the xQuest web interface (http://www.xquest.org) two times with search parameters fit for CID spectra of either the light or the heavy isotopes and two times with more restrictive parameters fit for HCD spectra of either the light or the heavy isotopes.  It was not necessary to presort the spectra into HCD and CID, because xQuest scores decrease when an HCD spectrum is evaluated with CID search parameters and vice versa thereby limiting the chance of false positives.  
The relaxed xQuest parameter set suited to CID spectra was (values adapted to the heavy isotope candidates in parentheses): cross-link mass-shift of 96.0211296 Da (100.0458296 Da), mono-link mass-shift of 114.0316942 Da  (118.0563942 Da), isotopic shift of 4.0247 Da (which had no effect on scoring because light and heavy peaks were submitted separately), MS1 tolerance of 5 ppm, MS/MS tolerance of 0.2 Da for normal fragment ions and 0.3 Da for cross-link fragment ions, electrospray ionization mode, absolute threshold for peak intensity of 1, fragment ion size (M/z) between 200 and 1600 Da, matching b and y ions only, trypsin as enzyme, maximum of two missed cleavages (cut at modified residues not allowed), cysteine carbamidomethylation as fixed modification, methionine oxidation as variable modification and a peptide length between 3 and 30 amino acids.  Both lysine and methionine were set as reactive amino acids — the latter just as a work-around to search for modified protein N-termini; methionine cross-link hits, in which methionine was not located at the N-terminus of the protein, were discarded later.  The stricter parameter set for HCD spectra differed from the former in the following way: MS/MS tolerance of 0.03 Da for normal fragment ions and 0.045 Da for cross-link fragment ions, absolute threshold for peak intensity of 400, and a fragment ion size (M/z) between 100 and 3000 Da.  Unfortunately ions with a single charge are not matched by the xQuest algorithm, although they were present in the spectrum.  However, this does not lead to the discovery of false positives as the score is decreased by unmatched MS/MS peaks rather than increased.  The xQuest score cut-off was 15 with both CID and HCD spectra.

Determination of acetylation rate
MS data was searched with the MASCOT algorithm (www.matrixscience.com) to identify chymotryptic cleavage products and acetyl modifications.  Basic search parameters were MS accuracy of 2 ppm, MS/MS accuracy of 0.6 Da, no enzyme and carbamidomethylation of cysteines as fixed modification.  Lysine acetylation and methionine oxidation were set as variable modifications and MS/MS spectra for both fragmentation techniques were searched using the same parameter set.  Peptides which include a lysine residue and were identified in both forms, i.e. with and without acetyl label, were selected for manual semi-quantification.  Mass traces for doubly and triply charged ions were extracted from MS survey scans in a 3 ppm window and peak areas were manually integrated.  Out of the resulting peak area the ratio of acetylated to non acetylated peptides was calculated.

Model building
Atomic models of InvG and PrgK domains were generated by template based homology modeling applying EscC and EscJ as templates and using the SWISS-MODEL Server (http://swissmodel.expasy.org/)3.  PrgK was further assembled into a 24-mer ring-structure based on the observed crystallographic contacts within the superhelical arrangement of EscJ monomers in the crystal without helical rise. Note that in EscJ the amino acid stretch from Asn134 to Gln≈139 is not resolved in the X-ray structure, however, the corresponding amino acids in PrgK (Asp133 to Lys144) have been modeled using the the SWISS-MODEL server, indicating a possible conformation of this amino acid stretch (marked with an *).  This domain is shown with a 70% transparency setting in Fig S7 (marked with an *).


Acknowledgements
Molecular graphics images were produced using the UCSF Chimera package 4 from the Resource for Biocomputing, Visualization, and Informatics at the University of California, San Francisco (supported by NIH P41 RR-01081).

References

1.	Mitulovic, G. et al. Preventing carryover of peptides and proteins in nano LC-MS separations. Anal Chem 81, 5955-5960 (2009).
2.	Rinner, O. et al. Identification of cross-linked peptides from large sequence databases. Nat Methods 5, 315-318 (2008).
3.	Arnold, K., Bordoli, L., Kopp, J. & Schwede, T. The SWISS-MODEL workspace: a web-based environment for protein structure homology modelling. Bioinformatics 22, 195-201 (2006).
4.	Pettersen, E. F. et al. UCSF Chimera--a visualization system for exploratory research and analysis. J Comput Chem 25, 1605-1612 (2004).
