# Supplementary figures and images for: Topology and Organization of the Salmonella typhimurium Type III Secretion Needle Complex Components
Source: PLoS Pathog. 2010 Apr 1;6(4):e1000824. doi: 10.1371/journal.ppat.1000824 (PMC2848554; doi:10.1371/journal.ppat.1000824)

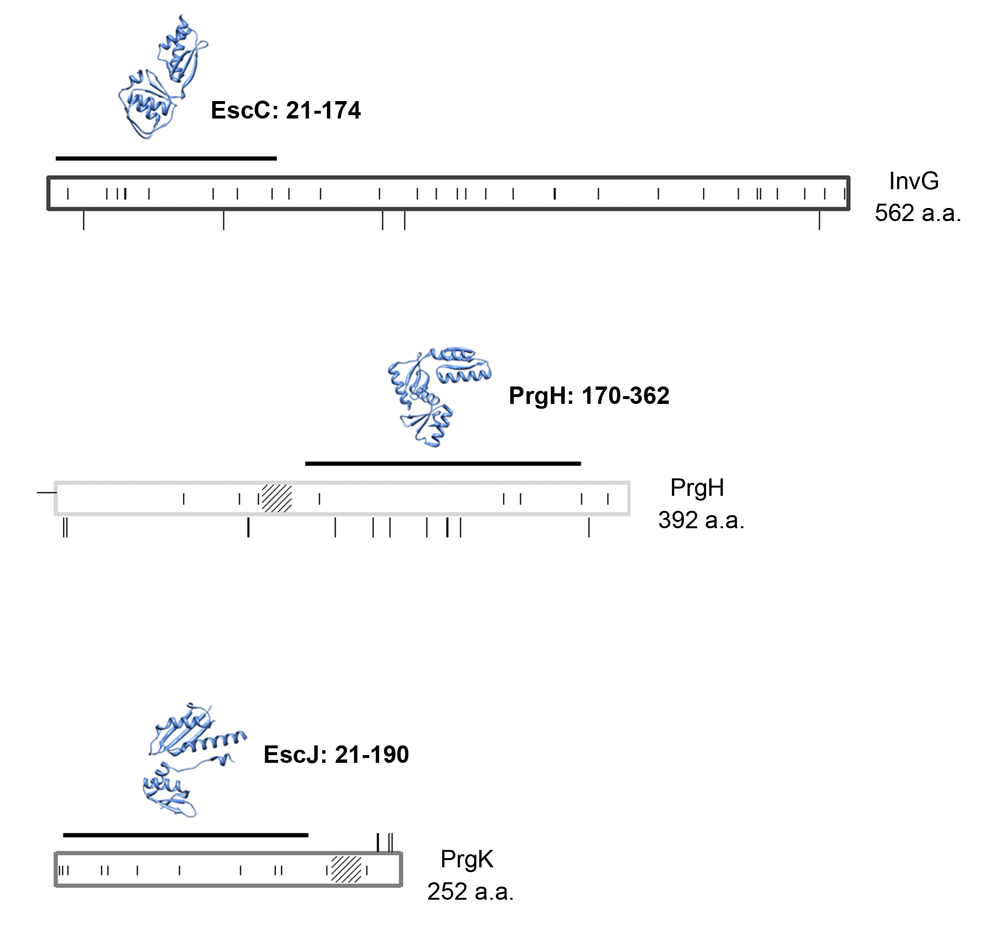

Supplement: Figure S1 — Block diagram of the three major base proteins, InvG, PrgH, and PrgK of the needle complex and region of atomic structures of PrgH and the homologues EscC (InvG) and EscJ solved. Vertical lines extending from the block diagram are positions of lysines that can be chemically derivatized and are presumably surface exposed. Remaining non-derivatizable (and probably not surface exposed) lysines are indicated as vertical lines within the block diagram.. Amino acid position for InvG, PrgH, and PrgK are indicated for the full length proteins prior signal peptide cleavage (processed mature InvG starts at Ser-25, and PrgK starts at Cys-18, respectively). (2.84 MB TIF) [file ppat.1000824.s002.tif]

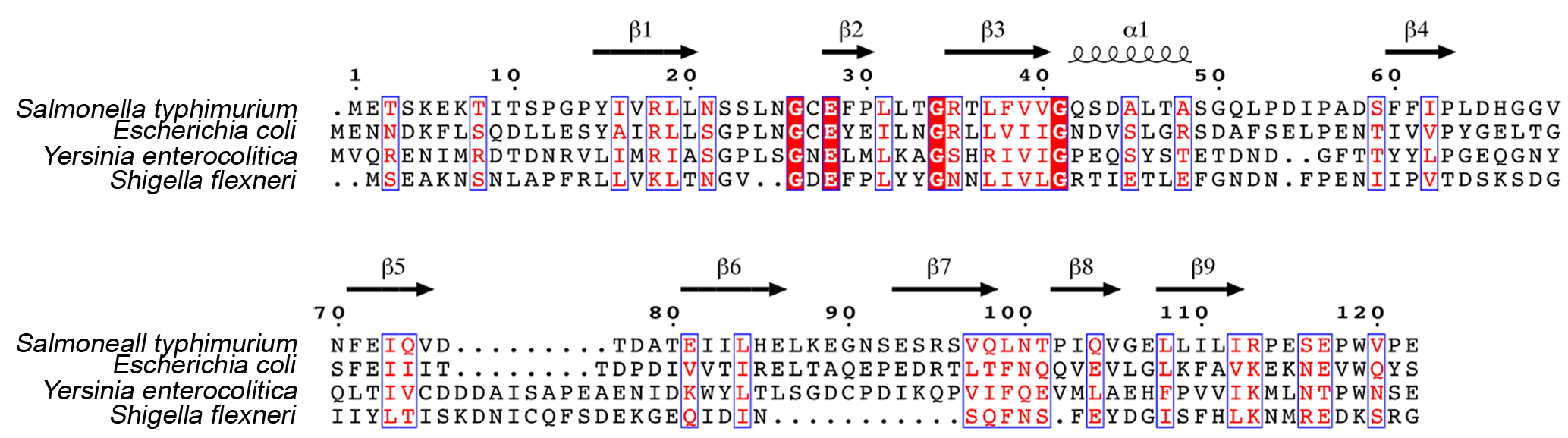

Supplement: Figure S2 — Sequence alignment and secondary structure prediction of the major part of the N-terminal domain of PrgH (1–122) from various species (S. typhimurium (PrgH), E. coli (EprH), Y. enterocolitica (Ye3550), S. flexneri (MxiG)). The N-terminal domain of PrgH is predicted to be mostly composed of beta-strands. Highly conserved and similar residues are indicated in red. (0.47 MB TIF) [file ppat.1000824.s003.tif]

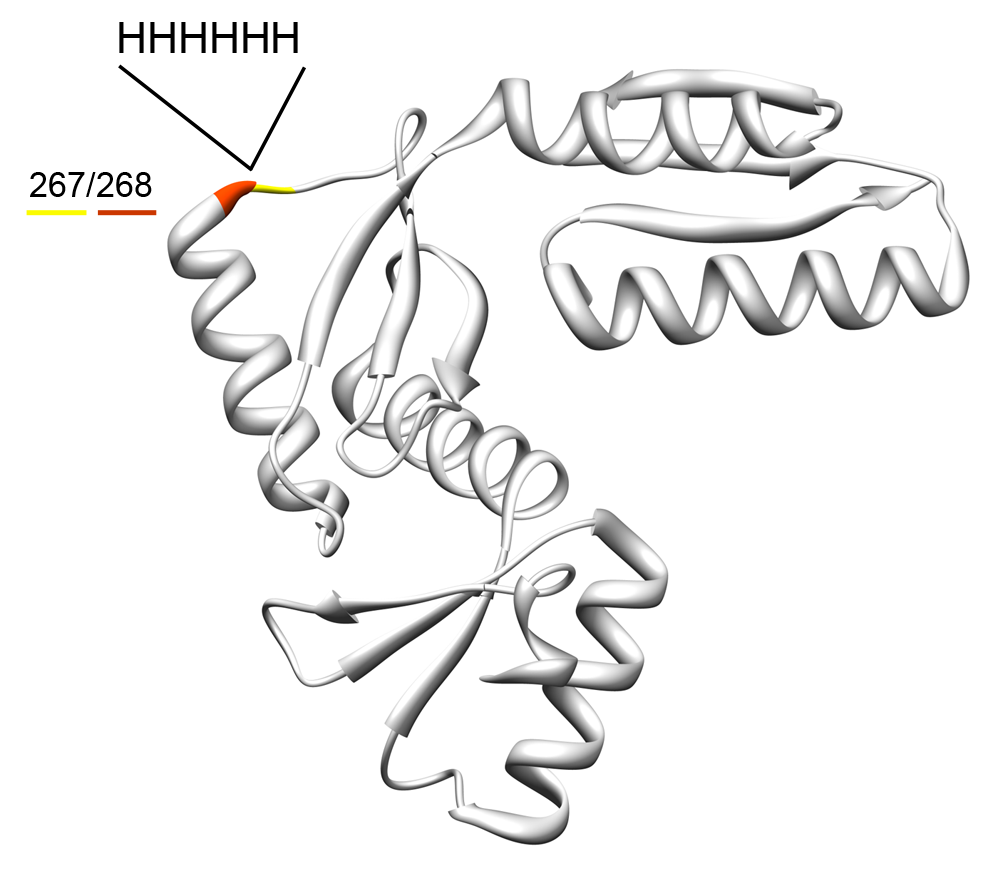

Supplement: Figure S3 — Ribbon diagram of PrgH (177–362) and position for insertion of a poly-histidine tag following amino acid 267 for Ni-NTA-nanogold labeling. (0.23 MB TIF) [file ppat.1000824.s004.tif]

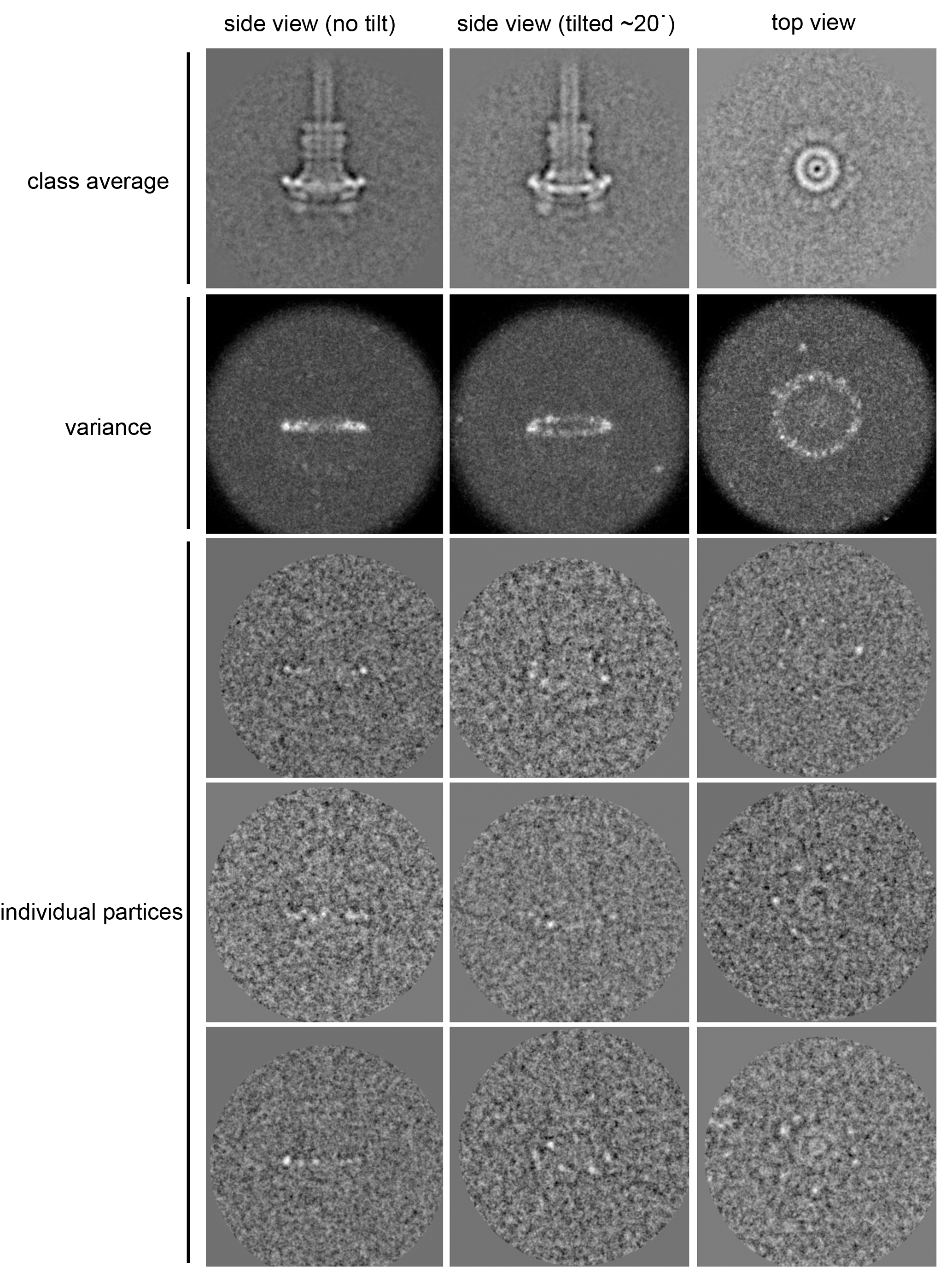

Supplement: Figure S4 — Single particle analysis of Ni-NTA labeled needle complexes with a poly-histidine insertion following position 267 in PrgH. Representation of various views of class-averages, variance-images, and individual particles of PrgH-267his labeled needle complexes show the presence of the nanogold label at the outermost perimeter of the inner rings. The highest variance was observed at the position of the nanogold label, which indicates that the individual particles are not uniformly labeled. This is also evident, when individual particles are visualized. (4.35 MB TIF) [file ppat.1000824.s005.tif]

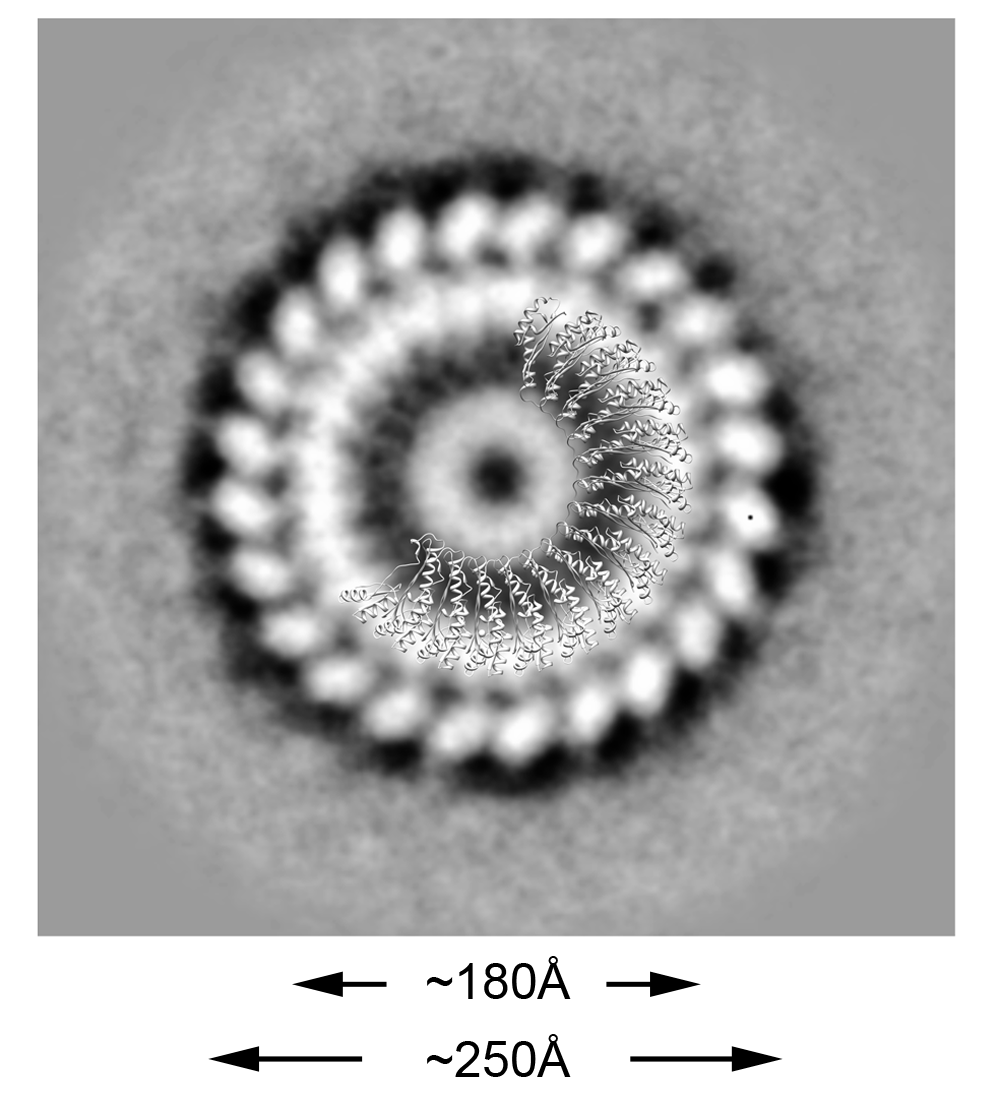

Supplement: Figure S5 — PrgK resembles the smaller concentric ring of the PrgHΔ4 inner ring substructure. En face view of the most prominent class-average derived after hierarchical clustering from images of negatively stained inner rings substructures from PrgHΔ4 complexes. The smaller concentric ring shares similarity in dimension (∼180Å) and organization to the modeled PrgK ring. In order to allow a comparison between the class average of the inner ring substructure and the modeled PrgK ring, only a segment of the latter is shown. (0.53 MB TIF) [file ppat.1000824.s006.tif]

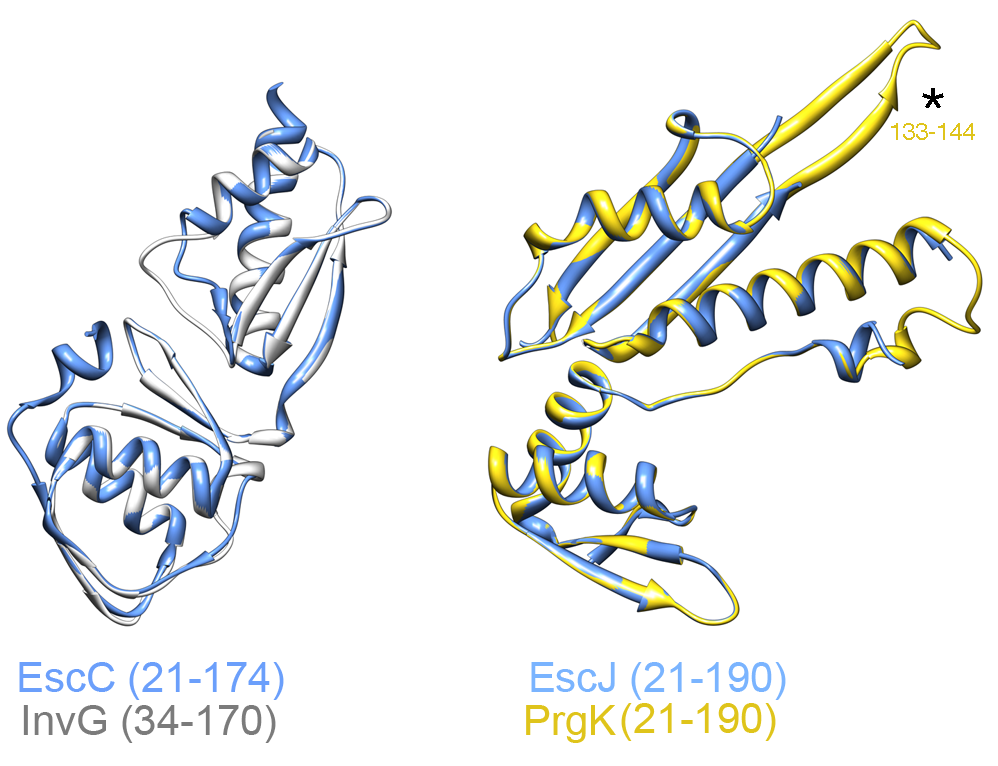

Supplement: Figure S6 — Ribbon diagram of modeled InvG and PrgK domains. The structure of InvG (white) and PrgK (yellow) were obtained by structure homology-modeling based on EscC (blue) and EscJ (blue) templates, using the SWISS-MODEL server (http://swissmodel.expasy.org/). Note that in EscJ Asn134 to Gln139 is not resolved in the X-ray structure, however, the corresponding amino acids in PrgK (Asp133 to Lys144) have been modeled using the the SWISS-MODEL server, indicating a possible conformation of this amino acid stretch (marked with an *). This domain (also marked with an *) is shown with a 70% transparency setting in Fig. S7. (0.43 MB TIF) [file ppat.1000824.s007.tif]

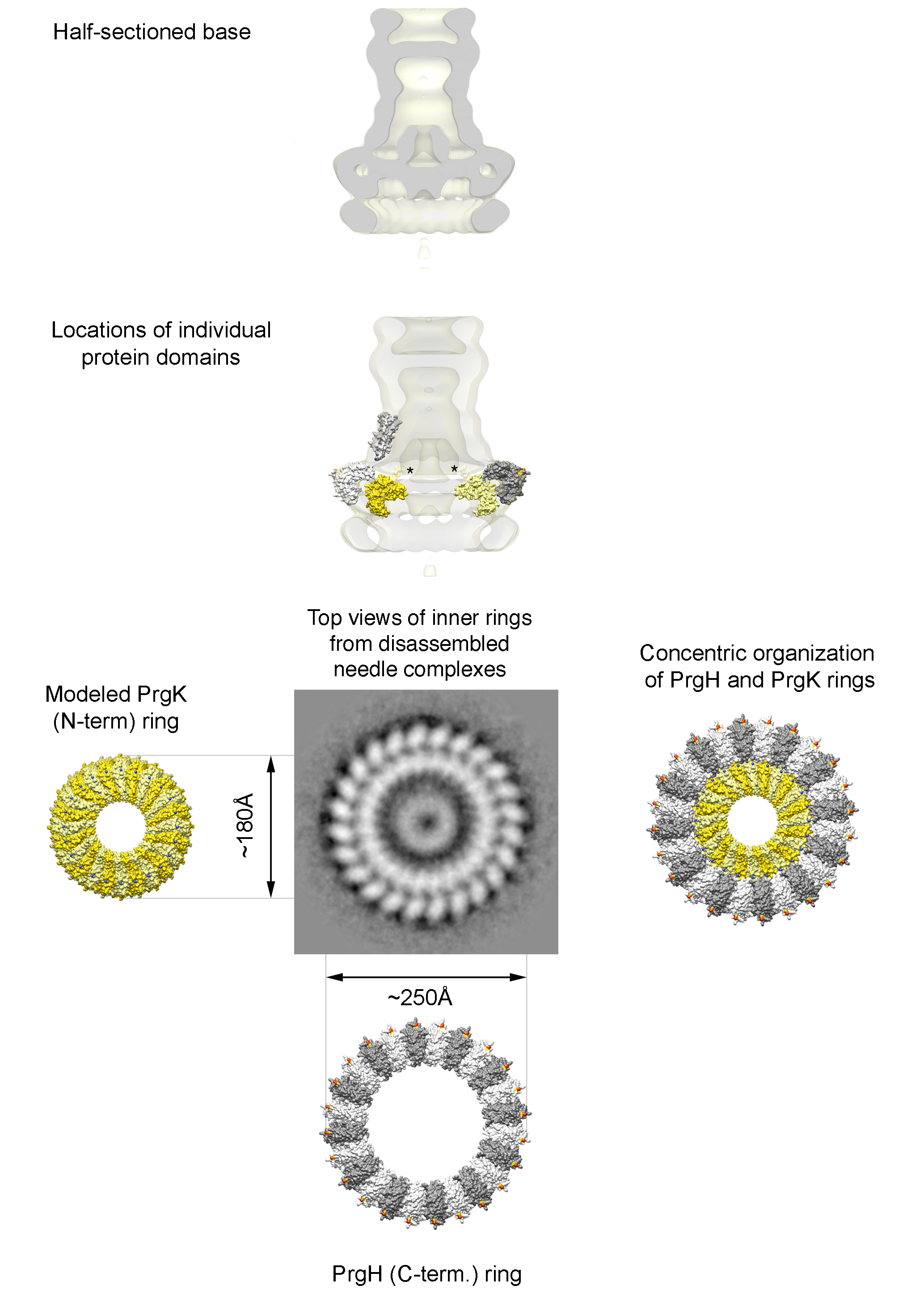

Supplement: Figure S7 — Overview of needle complex substructures and organization. Side view of half-sectioned base and location of individual protein domain. (* indicates to a possible confirmation of amino acids 133–144 in PrgK based on modeling using the SWISS-MODEL server (Fig. S6). Concentric ring-organization of IR1/2 revealed from top-viewed disassembled complexes and ring models of PrgH and PrgK. (1.48 MB TIF) [file ppat.1000824.s008.tif]
