# Supplementary material for: Topology and Organization of the Salmonella typhimurium Type III Secretion Needle Complex Components
Source: PLoS Pathog. 2010 Apr 1;6(4):e1000824. doi: 10.1371/journal.ppat.1000824 (PMC2848554; doi:10.1371/journal.ppat.1000824)
Supplement: Table S1 — Comparison of surface accessibility in wild type and ΔinvG mutant complexes. Peptides from NHS-acetate derivatized complexes were identified by MS/MS sequencing and semi-quantified by MS peak integration (Text S1). The table displays the modified lysine (Lysine), the identified corresponding peptide (Peptide) and the degree of NHS-acetate modification for the wild type (%w.t.) and the ΔinvG mutant (%ΔinvG). The ratio of peptide acetylation is an average of two independent mass spectrometry measurements. To highlight which lysines get stronger modified in the ΔinvG mutant (and are most likely more surface exposed) the ratio of ΔinvG mutant and wild type acetylation is determined (%ΔinvG/%w.t.). Due to the presence of several lysines within the peptides “K48, K53” and the C-terminally located peptides (“C-term” = K238, K239, K246, K248) the exact position of the derivatization could not be distinguished. (0.07 MB RTF) [file ppat.1000824.s009.rtf]

Supplementary Table S1: Comparison of surface accessibility in wild type and ∆invG mutant complexes.  

					
Lysine	Peptide	%w.t.	%Δ¤invG	%Δ¤invG/%w.t.	
					
					
K48,K53	QMHNIEANKIDSGKL	29.6	33.0	1.11	
K48,K53	QMHNIEANKIDSGKLGY	24.3	38.7	1.59	
K48,K53	MHNIEANKIDSGKLGY	29.1	31.1	1.07	
K48,K53	KIDSGKLGY	12.4	18.9	1.52	
K144	DIDAGENGRPPKPVHL	1.8	4.6	2.59	
K144	ISYDIDAGENGRPPKPVHL	4.5	7.5	1.68	
K168	AHQISDIKRF	0.3	5.6	21.77	
K168	AHQISDIKRFL	1.1	18.4	17.25	
K203	QAPGTPVKRN	7.5	11.1	1.48	
K203	QAPGTPVKRNSF	10.0	14.3	1.42	
K231	NSFATSWIVLIILLSVMSAGFGVWYYKNHYAR	23.5	29.7	1.26	
C-Term	ARNKKGITADDKAK	31.7	35.6	1.12	
C-Term	ARNKKGITADDKAKSSNE	50.0	43.2	0.86	
C-Term	KKGITADDKAKSSNE	76.4	64.3	0.84	
					

Peptides from NHS-acetate derivatized complexes were identified by MS/MS sequencing and semi-quantified by MS peak integration (Supplementary Protocol).  The table displays the modified lysine (Lysine), the identified corresponding peptide (Peptide) and the degree of NHS-acetate modification for the wild type (%w.t.) and the ΔinvG mutant (%ΔinvG).  The ratio of peptide acetylation is an average of two independent mass spectrometry measurements.  To highlight which lysines get stronger modified in the ΔinvG mutant (and are most likely more surface exposed) the ratio of ΔinvG mutant and wild type acetylation is determined (%ΔinvG/%w.t.).  Due to the presence of several lysines within the peptides “K48, K53” and the C-terminally located peptides (“C-term” = K238, K239, K246, K248) the exact position of the derivatization could not be distinguished.
