# Supplementary material for: Topology and Organization of the Salmonella typhimurium Type III Secretion Needle Complex Components
Source: PLoS Pathog. 2010 Apr 1;6(4):e1000824. doi: 10.1371/journal.ppat.1000824 (PMC2848554; doi:10.1371/journal.ppat.1000824)
Supplement: Table S2 — List of mono-links and loop-links determined by chemical derivatization and mass spectrometry. In order to specify all lysines being accessible for derivatization, hetero-cross-links (cross-link between two different proteins, see Table 1), mono-links (modified peptides that reacted with only one functional group of the bivalent cross-linker (BS2G (0/d4))) and loop-links (cross-links within one polypeptide chain or between homo-proteins) were determined. Only peptides with an xQuest score of higher than 15 are displayed as those cross-linking results are likely to be reliable (see Text S1). The respective modified amino acid is displayed in bold. Under “Monolink/Looplink” listed positions are the absolute positions of the respective protein (including signal peptides), “Error” the deviation of measured precursor ion mass from theoretical precursor ion mass in parts per million, and “Score” the xQuest score. (0.10 MB RTF) [file ppat.1000824.s010.rtf]

Supplementary Table S2: List of mono-links and loop-links determined by chemical derivatization and mass spectrometry.  

				
Monolink/ Looplink	Peptide	Mass Error (ppm)	xQuest Score	
				
				
InvG				
K38	IPVTGSGFVAKDDSLR	0.6	21.9	
K134	SGLYNKNYPLR	1.0	18.1	
K244	GKAANYAGGMSLQEALK	0.7	34.2	
K244-K259	GKAANYAGGMSLQEALK	0.5	18.2	
K545	QSGAWSGDDKLQK	0.6	15.6	
				
PrgH				
N-K7	METSKEK	1.2	18.7	
K5	METSKEK	1.3	17.2	
K7	EKTITSPGPYIVR	1.2	30.1	
K132-K133	LETSAKKNEPR	0.8	18.5	
K133	LETSAKKNEPR	1.3	16.5	
K192	DKMLYVAAQNER	0.8	26.0	
K218	GDYDKNAR	1.5	31.3	
K230	VINENEENKR	0.7	19.5	
K255	KPVFWLSR	0.9	31.7	
K269-K270	NTMSKKELEVLSQK	0.1	20.7	
K270	KELEVLSQK	0.5	31.0	
K278	ELEVLSQKLR	1.6	20.6	
K278	KELEVLSQK	0.5	16.7	
K278	KELEVLSQKLR	0.9	19.5	
				
				
PrgK				
				
K238-K239	NKKGITADDK	1.3	31.7	
K239	KGITADDK	1.6	33.3	
K239	KGITADDKAK	1.1	21.6	
K239-K248	KGITADDKAK	1.6	23.5	
K246	KGITADDK	2.0	23.6	
K246	GITADDKAK	2.0	26.1	
K246	GITADDKAKSSNE	0.9	26.8	
K246-K248	GITADDKAKSSNE	2.3	25.3	
K248	GITADDKAKSSNE	0.7	23.5	
				

In order to specify all lysines being accessible for derivatization, hetero-cross-links (cross-link between two different proteins, see Table 1), mono-links (modified peptides that reacted with only one functional group of the bivalent cross-linker (BS2G (0/d4))) and loop-links (cross-links within one polypeptide chain or between homo-proteins) were determined.
Only peptides with an xQuest score of higher than 15 are displayed as those cross-linking results are likely to be reliable (see Supplementary Protocol).  The respective modified amino acid is displayed in bold.  Under “Monolink/Looplink” listed positions are the absolute positions of the respective protein (including signal peptides), “Error” the deviation of measured precursor ion mass from theoretical precursor ion mass in parts per million, and “Score” the xQuest score.
